# The influence of arm positions on abdominal image quality of whole-body computed tomography in trauma: systematic review

**DOI:** 10.1007/s10140-019-01732-w

**Published:** 2019-11-27

**Authors:** E. S. Speelman, B. Brocx, J. E. Wilbers, M. J. de Bie, O. Ivashchenko, Y. Tank, A. J. van der Molen

**Affiliations:** 1 Student Clinical Technology, Faculty of Mechanical, Maritime and Materials Engineering (3mE), Mekelweg 2, 2628 CD Delft, The Netherlands; 2 Department of Radiology, C-2S, Leiden University Medical Centre, Albinusdreef 2, 2333 ZA Leiden, The Netherlands

**Keywords:** Tomography, X-ray computed, Traumatology, Patient positioning, Artifacts, Systematic review

## Abstract

**Purpose:**

Whole-body computed tomography (WBCT) is the standard diagnostic method for evaluating polytrauma patients. When patients are unable to elevate their arms, the arms are placed along the body, which affects the image quality negatively. Aim of this systematic review is to evaluate the influence of below the shoulder arm positions on image quality of WBCT.

**Methods:**

Literature in PubMed and Scopus databases was systematically searched. Results of the papers were stratified into 4 categories: arms elevated, 1 arm up 1 arm down, arms ventrally supported, arms along the body. A qualitative analysis was performed on subjective image quality and a quantitative analysis on objective quality (image noise).

**Results:**

Eight studies were included with 1421 participants. Various studies reported significantly higher quality scores with arms elevated, compared to arms along the body. Significant differences in objective image quality were found between the arms elevated and the arms ventrally on support group. The arms ventrally supported group had a significantly higher image quality than the arms along the body group. A statistically significant difference was found in objective image quality between the 1 arm up 1 arm down and arms along the body group. No preferential below the shoulders position could be identified.

**Conclusion:**

Positioning the arms alongside the body results in a poor image quality. Placing the arms on a pillow ventrally to the chest improves image quality. Interestingly, asymmetrical arm positioning has potential to improve the image quality for patients that are unable to elevate the arms.

## Introduction

**Fig. 1:**
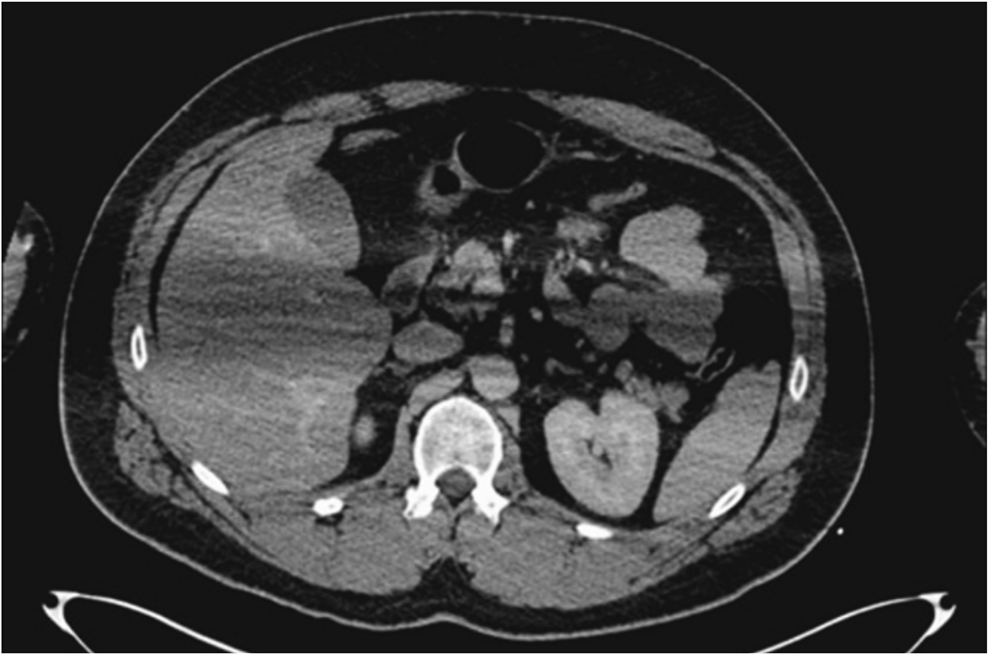
Illustration of image quality degradation due to arm-related beam hardening (Karlo et al. [12])

In 2010, trauma caused 5,073,300 deaths worldwide among all ages [1]. Among persons from 15 to 19 years of age, trauma is the leading cause of death, and in the Netherlands, 19.1% of all polytrauma patients who were admitted in the hospital died [2, 3]. Therefore, it is necessary that polytrauma patients receive fast and accurate care. Whole-body computed tomography (WBCT) makes this possible [4]. WBCT is recommended as a standard and basic diagnostic method for trauma patients [5, 6]. The standard protocol of the WBCT is divided in two stages. First, positioning the arms at the patient’s side for head and neck CT, and then repositioning the arms up, in order to maintain the best image quality for thoraco-abdominal CT [7]. However, in case of fractures or dislocations of shoulders or arms, patients may be unable to elevate the arms and need to be imaged with the arms alongside the body. This position can hamper image quality of chest and abdomen CT images as a symmetric position of arms in the same plane as the organs of interest (e.g. heart, lungs, liver, spleen) results in an artefact ‘bar’ across the CT images (Fig. 1). It may result in misdiagnosis, ultimately compromising correct treatment of the patient [8, 9].

The aim of this systematic review is to evaluate the influence of different (below the shoulder) arm positions on image quality of WBCT for polytrauma patients.

## Methods

A systematic review was performed in order to investigate the influence of arm positions on image quality during the image acquisition in WBCT. The Preferred Reporting Items for Systematic Reviews and Meta-Analyses (PRISMA) guidelines were followed [10].

### Search strategy

Data was collected from the PubMed database, searching with the MeSH keywords ‘Tomography, X-Ray Computed’, ‘Arm’, ‘Whole Body Imaging’, ‘Traumatology’. The Scopus database was searched with similar keywords. The complete search strings are included in Appendix 1. PubMed was searched on May 2nd, and Scopus on May 9th of 2019. Articles from the past 20 years were included, considering the rapid improvements in CT technology.

### Exclusion criteria and quality assessment

Firstly, the articles were screened on title and abstract. In order to assess the relevance of the articles, exclusion criteria were established in consensus. Articles were excluded if they were a review, comment, or letter to the editor, did not cover WBCT, did not investigate arm positioning, or did not observe a human adult population (i.e. 18 years and older).

Secondly, the full text articles were examined. Articles that did not mention image quality as outcome measure, did not investigate trauma patients, or were not available in English, Dutch, or German were excluded. Exclusion was performed by four observers. Articles were randomly distributed, so that every article was seen by two observers.

Finally, a qualitative research level assessment was performed on the remaining articles, in order to determine the quality of the articles. Based on assessment criteria listed in Table 1, quality scores (i.e. number of stars) were independently assigned by two observers. Doubts or disagreements were resolved through discussion with all four observers. Articles with a score below four stars were excluded.

**Table 1:** Quality assessment criteria

Criteria	Points
Selection	
Subject inclusion criteria	
Inclusion criteria are reproducible.	✶
No description.	
Selection of the non-intervention cohort	
Drawn from the same community as the intervention cohort.	✶
Drawn from a different source.	
No description.	
Outcome measurement	
Assessment of outcome	
Independent blind assessment is performed.	✶
In case of objective measurement, the methods are reproducible.	✶
None of the above.	
Assessment of subjects	
From all included subjects, results are derived.	✶
Subjects are lost without description.	
Comparability	
Comparability on the basis of the design	
Difference(s) in characteristics is/are defined (e.g. age, sex).	✶
No description.	
Comparability on the basis of the cohort	
Study controls for possible confounders.	✶
Not performed or no description.	
Comparability in CT settings	
Same CT-settings are executed in all groups.	✶
Study controls for different CT-settings.	✶
No control or no description.	

Qualitative analysis was performed on each included article. Selection, outcome measurement, and comparability were main stakeholders. A maximum score of 7 points could be derived when an article meets all statements

### Data analysis and outcomes

Data were independently analysed by four researchers. Image quality was divided in a subjective and objective component. Subjective image quality was extracted if measured with a Likert scale. Objective image quality measured as image noise in Hounsfield Units (HU) was extracted. The subjective and objective image quality were independently assigned as outcomes.

### Statistical analysis

Due to high discrepancy of subjective image quality reporting parameters found in the literature, a qualitative analysis was performed for this outcome. For the objective image quality, a quantitative analysis was performed on image noise.

## Results

### Study selection

**Fig. 2:**
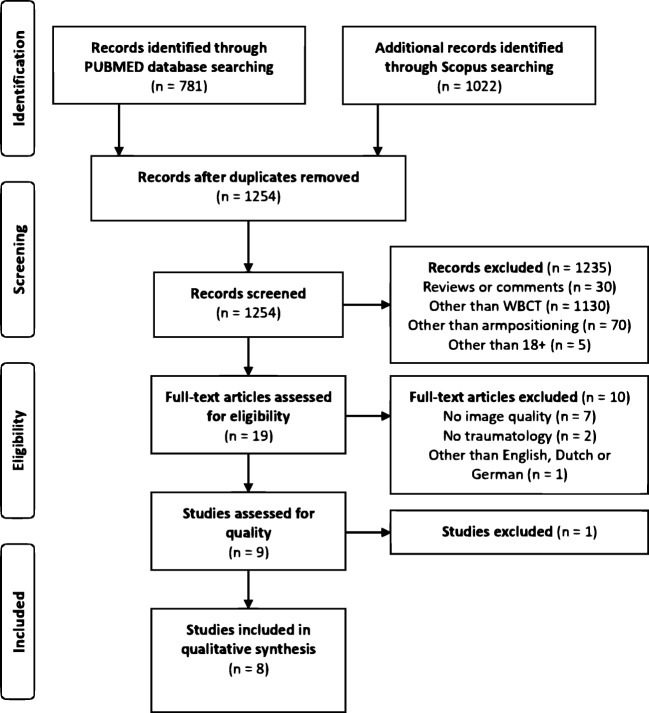
Study inclusion flow diagram

On May 2, 2019, the database search derived a total of 1254 articles. In the first round of exclusion, a total of 1234 articles were excluded because they were reviews, comments, or letters to the editor (*n* = 30), concerned another imaging modality than WBCT (*n* = 1130), did not use arm positioning as an intervention (*n* = 70), or did not include patients over 18 years of age (*n* = 4). A total of nine articles was excluded based on full text assessment. After the quality assessment, one article was excluded [11], and eight articles were found eligible for this review and were therefore included (Fig. 2) [8, 9, 12–17].

### Quality assessment

All articles were assessed on quality, deriving a mean score of five (Appendix 2, Table 6). One article was excluded based on a poor quality assessment (score 3).

### Study characteristics

A total of 1421 subjects were examined in all the studies. Six studies were retrospective and two were prospective. The study characteristics are summarised in Appendix 2, Table 7. Three studies assessed both subjective and objective image quality, two studies assessed only objective image quality, and three studies assessed only subjective image quality. Subjective and objective outcome measures are summarised in Tables 2 and 3, respectively. Studies examined similar arm positions. These arm positions are grouped and labelled.

**Table 2:** Subjective measurements of image quality

Study	Outcome	Organs
Beenen et al. [13]	5-point scale 1 = non-diagnostic image quality 2 = poor image quality 3 = satisfactory image quality 4 = good image quality 5 = excellent image quality	Overall quality of the total body CT Brain Cervical spine Thoracolumbar spine Lung parenchyma Mediastinum Liver Spleen Kidney Pelvis Aortic arch Portal vein Abdominal aorta at the level of the superior mesenteric artery
Brink et al. [14]	5-point scale 1 = non-diagnostic image quality 2 = poor image quality 3 = fair image quality 4 = good image quality 5 = excellent image quality	Aorta Liver Spleen Kidneys Spine Pelvis
Hickethier et al. [15]	3-point scale 1 = excellent image quality, no artefacts 2 = diagnostic image quality, artefacts present 3 = non-diagnostic image quality due to severe artefacts	Lungs Aorta Liver Spleen Thoracoabdominal spine
Hoppe et al. [8]	3-point scale 0 = no substantial artefacts 1 = moderate artefacts^a^ 2 = major artefacts^b^	Liver Spleen
Kahn et al. [9]	4-point scale 1 = no artefact, excellent image quality 2 = minor artefact, no relevant effect on image quality 3 = moderate artefact with degradation of image quality^c^ 4 = severe artefact, precluding reliable image interpretation	Liver Spleen Kidneys Pelvis Mean of all organs

Overview of subjective outcome measurements of image quality and evaluated organs. Sorted by study

^a^Not impairing a diagnostic evaluation of liver and spleen

^b^Impairing a diagnostic evaluation and necessitating a repeat scan with raised arms

^c^Still adequate diagnostic quality

**Table 3:** Objective measurements of image quality

Study	Outcome	Organs
Beenen et al. [13]	Contrast enhancement (HU)	Aortic arch Abdominal aorta Portal vein Parenchym liver Parenchym spleen Parenchym renal cortex
Brink et al. [14]	Image noise	Liver parenchym at the level of the porta hepatis in liver segment VI or VII.
Karlo et al. [12]	Image noise	Liver segment VI or VII
Reske et al. [16]	Image noise	Liver segment VI Aorta
Sedlic et al. [17]	Image noise	Aorta

This table summarises the objective measurements of image quality used in the included articles. Image noise is defined as the mean standard deviations (SD) of CT pixel values in Hounsfield Units (HU)

Study results were stratified into the following groups according to arm position:

- Position A: arm repositioning (both arms elevated for thoraco-abdominal CT and arms down for head and neck CT) *and* both arms elevated (alongside the head)
- Position B: one arm elevated and one arm either down, *or* in front of the upper abdomen, *or* in front of the pelvic area
- Position C: arms on a pillow ventrally to the chest or upper abdomen *and* both arms crossed in front of the upper abdomen (without pillow)
- Position D: both arms down (alongside the body) *and* both arms crossed in front of the pelvic area

Arm positions A and D were compared eight times, from which five times subjectively and three times objectively (i.e. image noise). Arm positions A and C were compared four times, from which three subjectively and one objectively. The subjective image quality was twice compared for arm positions C and D and once objectively.

### Subjective image quality

Radiologists assessed the subjective image quality for designated organs. In all studies, the observers were blinded to the arm position through cropping of the images. Among all the included articles, for six organs, subjective image quality scores were reported for positions in group A and D. These findings are presented in Table 4. It shows if a study found a significant difference in image quality per organ based on the Likert scale.

**Table 4:** Subjective image quality arm positions A and D

No significant difference^a^		Organ	Significant difference^b^				
		Spleen	Beenen et al.	Brink et al.	Hoppe et al.	Kahn et al.	Karlo et al.
		Liver	Beenen et al.	Brink et al.	Hoppe et al.	Kahn et al.	Karlo et al.
	Beenen et al.	Kidneys	Brink et al.	Kahn et al.			
Beenen et al.	Brink et al.	Aorta	Karlo et al.				
	Beenen et al.	Spine	Karlo et al.				
Beenen et al.	Brink et al.	Pelvis	Kahn et al.				

Overview of differences in subjective image quality per organ. Compared for arm position A: arm repositioning (arms elevated for abdomen CT and arms down for head and neck CT) and both arms elevated (alongside the head), arm position D: arms down (alongside the body) and both arms crossed in front of the pelvic area. Names of authors refers to the used articles, Beenen et al. [13], Brink et al. [14], Hicktetier et al. [15], Hoppe et al. [8], Kahn et al. [9], and Karlo et al. [12]

^a^No significant difference between arm positions A and D in subjective image quality

^b^Significantly higher subjective image quality for arm position A, as compared to arm position D

Five studies found significantly higher quality scores for the spleen and liver with arm position A compared to D. Contradicting results have been found for the kidneys, aorta, spine, and pelvis. For example, Brink et al. [14] observed no significant effect in the regions of the aorta, while Karlo et al. [12] did.

Subjective image quality of arm positions in group A and C was evaluated several times for the spleen, liver, aorta, and spine. These findings are presented in Table 5.

**Table 5:** Overview of differences in subjective image quality per organ

No significant difference^a^	Organ	Significant difference^b^		
	Spleen	Hickethier et al.	Kahn et al.	Karlo et al.
	Liver	Kahn et al.	Karlo et al.	
	Aorta	Hickethier et al.	Karlo et al.	
Hickethier et al.	Spine	Karlo et al.		

Compared for arm position A: arm repositioning (arms elevated for abdomen CT and arms down for head and neck CT), arms alongside the head and C: arms on a pillow ventrally to the chest with both arms flexed at the elbow and the forearms positioned next to each other. Names of authors refers to the used articles, Hicktetier et al. [15], Kahn et al. [9], and Karlo et al. [12]

^a^No significant difference between arm positions A and C in subjective image quality

^b^Significantly higher subjective image quality for arm position A, as compared to arm position C

In the assessment of the subjective image quality for arm position C compared to arm position D, Karlo et al. [12] found a significantly higher image quality of the spleen, liver, and aorta (all, *p* = < 0.05). Kahn et al. [9] also found a significant difference. From the image quality in the liver, spleen, kidneys, and pelvis, a Mean of All Organs (MAO) was calculated. A statistically significant difference in the MAO was found between arm positions C and D (*p* = 0.029).

One study (Kahn et al. [9]) mentions two cases in which the WBCT did not reveal existing injuries, that were later on discovered through other imaging modalities. These injuries were missed in the CT scan due to artefact-related degradation of image quality.

### Objective image quality

Of the five studies evaluating objective image quality, four measured image noise. Three studies, that all measured this outcome in the hepatic region, were compared [12, 14, 16].

**Fig. 3:**
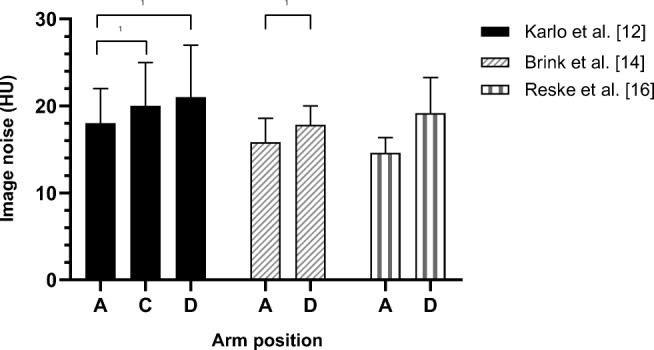
Image noise in hepatic region. Distribution of image noise in patients with different arm positions. Image noise is defined as the mean standard deviations (SD) of CT pixel values in Hounsfield Units (HU), measured as mean ± SD. Boxes represent means and whiskers represent SD. The groups with values significantly different from each other are indicated (✱). A: arm repositioning (arms elevated for abdomen CT and arms down for head and neck CT) and arms elevated (alongside the head), C: arms on a pillow ventrally to the chest or upper abdomen and both arms crossed in front of the upper abdomen, D: arms down (alongside the body) and both arms crossed in front of the pelvic area

To determine image noise within the region of interest (ROI), the mean standard deviations (SD) of CT pixel values in Hounsfield Units (HU) was reported. This mean ± SD was defined as image noise. All results of image noise per arm position are shown in Fig. 3. Significant differences were found between group A and D (Karlo et al. [12] and Brink et al. [14]) and between group A and C (Karlo et al. [12]) (*p* < 0.05 and *p* < 0.006, respectively).

Reske et al. [16] measured image noise in both mean ± SD and median ± range, although they did not perform a statistical analysis on the mean ± SD. On the median ± range, only non-parametric tests were performed. Here, a significantly better image quality was found in group A, compared to group D.

Karlo et al. [12] found no significant difference in image noise between arm positions C and D, as shown in Fig. 3.

### Individual findings

The comparison between arm positions A and B, and positions B and D, have only been made by individual studies. The results of these studies are discussed below.

Brink et al. [14] observed no significant effect in noise in the hepatic region between arm positions A and B (15.8 ± 2.8 HU vs. 16.6 ± 2.7 HU, respectively). Regarding subjective image quality, one radiologist judged the subjective image quality to be significantly lower in arm position B. However, the other radiologist did not find a significant difference between arm positions A and B.

The analysis of Kahn et al*.* [9] revealed a statistically significant difference in the MAO in subjective image quality between arm positions B and D, in favour of B (*p* = <0.001). The image quality of position B was also found to be statistically higher than arm position C (*p* = 0.01).

### Subjective and objective image quality

**Fig. 4:**
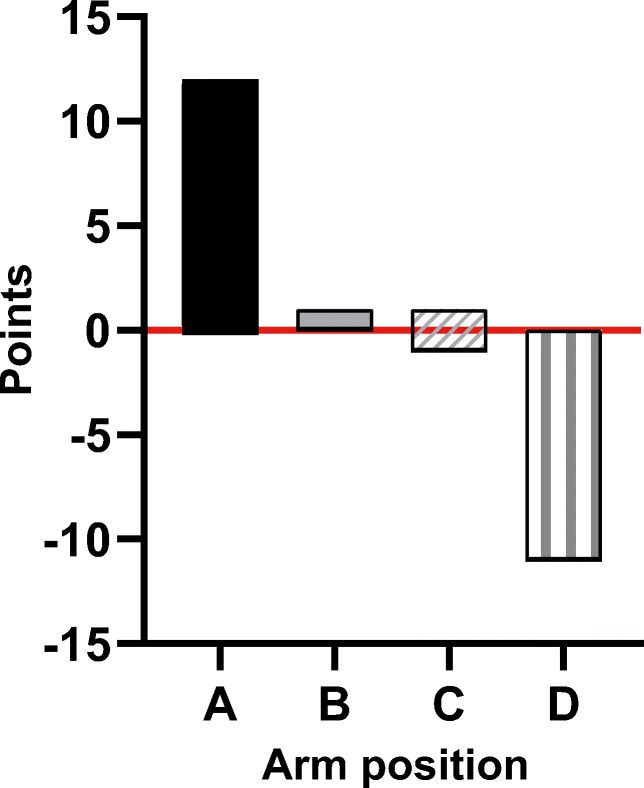
Overall results on image quality. Overview of the overall results of different arm positions. A point was assigned if an examined arm position had a significantly lower noise level or better subjective image quality. For the other examined arm position, a point was subtracted. No points were assigned when no significant difference was found. Arm positions, A: arm repositioning (arms elevated for abdomen CT and arms down for head and neck CT) and arms elevated (alongside the head), B: one arm elevated, and one arm down or in front of the upper abdomen or in front of the pelvic area, C: arms on a pillow ventrally to the chest or upper abdomen or in front of the pelvic area, D: arms down (alongside the body) and both arms crossed in front of the pelvic area

To get an overview of all the included articles, a point system was established. This visualises how often an arm position was evaluated and how often it was in advantage. For each comparison where an arm position had a significantly lower noise level or a significantly better subjective image quality, one point was assigned. For the other arm position, one point was subtracted. When no significant difference was found, no points were assigned. When subjective image quality was assessed by organ, the majority of outcomes of organs was evaluated. Figure 4 summarises these results.

## Discussion

This review demonstrated that positioning the arms in position A results in significantly lower image noise [12, 14, 16] (Fig. 3) and in significantly better subjective image quality of the liver and spleen [8, 9, 12, 14, 15] (Table 4) in comparison to arm position D. Hence, arm position A delivers the best image quality.

Furthermore, subjective image quality of the liver, spleen, and aorta is significantly lower when applying arm position C compared to arm position A [9, 12, 15] (Table 5).

Moreover, Karlo et al. [12] and Kahn et al. [9] found a significantly better subjective image quality of the liver and spleen in arm position C compared to D, thus sparing the upper abdomen from possible artefacts. Subsequently, positioning the arms on a pillow ventrally to the chest might be an alternative for patients with contraindications to elevate both arms.

Only individual studies evaluated the difference between arm positions B and D [9], B and C [9], and A and B [14], so no conclusive statement could be made. However, some interesting findings were reported.

Kahn et al. [9] found a significantly better subjective image quality in arm position B compared to D. Brink et al. [14] compared position A and B, but no significant difference was found. Further research is necessary to either accept these findings or to reject them. Nevertheless, it remains interesting for patients with one-sided trauma on the arms that one arm in the Field Of View (FOV) does not give significant artefacts, compared to no arm. Elevating one arm and leaving the other arm alongside the body asymmetrically might be a good method to improve image quality.

Additionally, Kahn et al. [9] evaluated the following four arm positions that were grouped under the position-groups (i.e. A, B, C, and D) of this article: both arms crossed in front of the upper abdomen, one arm placed in front of the upper abdomen, both arms crossed in front of the pelvic area and one arm placed in front of the pelvic area. A more elaborate evaluation of the interrelated results can be set out. Overall, elevation of one arm delivered significantly better image quality than placing both arms in the scan field. For patients that are unable to elevate one arm, it was found that for the best image quality, the one arm should be positioned on the upper abdomen. Placing one arm alongside the body resulted in the lowest image quality. When a patient is unable to elevate both arms, after placing the arms alongside the body, positioning the arms on the pelvis resulted in lowest image quality. To achieve best image quality, Kahn et al. [9] demonstrated that the arms should be crossed in front of the upper abdomen. Further research is necessary to confirm whether those arm positions make a good alternative for arm repositioning, for they seem very promising.

As demonstrated in this systematic review, arm position A has advantage in image quality over arm position D. Hoppe et al. [8] also contributed to this finding.

Additionally, Hoppe et al. [8] report an alternative arm position for patients that have a clinical contra-indication to elevate the arms above the head, e.g. due to fractures or dislocations. In such cases, the arms could be placed on foam sponge ramps by the patient’s side at a 25–30° angle, optionally asymmetrically in different angles. In a study, focusing on scan times, Ptak et al. [18] applied this positioning protocol to some patients that were unable to elevate the arms. The arms were placed at an angle, but non-parallel. More specific research is required to obtain results concerning image quality and make a comparison with a control group.

Our study has a number of limitations. The comparison performed by this systematic review was complicated by variations in CT settings, and the use of Automatic Exposure Control (AEC). It cannot be surely said that the CT parameters did not contribute to differences in image quality. Also, differences in scanning protocol were present. For example, the use of contrast varied. Another variation in study design was observed in the measurements of subjective image quality. Often, a variation of Likert scales was used (Table 2). Some studies used a 3-point scale, while others used a 5-point scale. This could affect the level of significance. In measuring image quality, some studies did not perform an inter-observer agreement correction. This could have had impact on the significance level of the subjective image quality. Also, group size was not similar among studies. The influence of this is unclear.

Moreover, in two studies [13, 16], an extra subgroup was established with a variation in radiation doses (i.e. similar arm positioning). In these cases, the data of the subgroup with the settings most similar to the control group of that study was extracted for analysis.

All included studies examined organs in the abdominal region. Therefore, in this review, the influence on these organs was compared. Consequently, the repositioning group (arms elevated for abdomen CT and arms down for head and neck CT) and arms elevated group were taken together as one (group A), because the arms were placed in the same area during the abdominal CT. Also, some studies (Sedlic et al. [17] and Hoppe et al. [8]) examined a group positioned with both arms up, but positioned the arms asymmetrically (one up and one down) when this was clinically not permissible because of trauma. In this review, these groups were placed in group A because the articles did not provide numbers of both positions.

Overall, in order to assign several positions to subgroups, some generalisation had to be made. This resulted in some heterogeneity in the subgroups. Another consequence was that for the study of Kahn et al. [9], various researched arm positions were placed in the same group of this study. At some instances, this resulted in multiple numerical results for one arm position. In these cases, this is resolved by taking the mean.

An important statement that was twice made on subjective image quality concerned diagnostic functionality [13, 15]. In both instances, it was emphasised that the image quality remained within diagnostic limits, while a low subjective image quality was found. Therefore, the results of this review concerning significant differences in image quality should be interpreted with caution. It can be the case that, even with a lower image quality, the diagnostic quality is still sufficient.

Sedlic et al. [17] compared two different WBCT protocols. In one protocol, the arms are positioned up if clinically permissible, and in the other protocol, the arms were elevated above the head for the CT scan of the abdomen and pelvis, and the arms were down for the CT scan of the neck and chest. Both groups would therefore be placed in group A, and therefore, no comparison could be made. Secondly, the image noise parameters that were measured in this study were measured in the thoracic region. Another study, Reske et al. [16], also measured image noise in the aorta, but at the level of the upper abdomen. Therefore, these two studies with different regions of interest were therefore left out in the analysis. Beenen et al. [13] only assigned objective image quality by HU attenuation and could therefore not be compared to the other studies.

An outcome that was often measured by the articles was the number of artefacts. However, these artefacts can be caused by different reasons. These are for instance incidental movement artefacts, breathing artefacts, patient positioning artefacts, or foreign objects within the FOV (i.e. metal) artefacts. Not all studies show the cause of the artefacts they mention. As a result, it is not known whether the artefacts found in the studies are caused by positioning of the arms or not.

Another limitation of this review is that, while one study mentioned missed injuries [9], an overview of non-diagnostic scans caused by certain arm positions is absent due to a lack of data. Some articles reported cases of ‘severe artefacts’ or ‘major artefacts’. The assumption could be made that these cases were necessitating a repeat scan, but this is not confirmed by the authors.

In conclusion, this systematic review set out various arm positions and their influence on image quality. As expected, positioning the arms alongside the body results in a poor image quality. However, positioning the arms on a pillow ventrally to the chest may improve image quality compared to positioning arms alongside the body. Interestingly, asymmetrical arm positioning has potential to improve the image quality for patients that are unable to elevate the arms. Therefore, more research is required to establish a decisive advantage on image quality.

## Appendix 1. PubMed and Scopus search string

PubMed:

(“Tomography, X-Ray Computed”[Mesh] OR “x-ray”[tw] OR “x-rays”[tw] OR computed tomograph*[tw] OR “CT”[ti]) AND (“Arm”[Mesh] OR “Arm”[tw] OR “Arms”[tw]) AND (“Whole Body Imaging”[Mesh] OR “Whole Body”[tw] OR “total body”[tw] OR “Traumatology”[Mesh] OR “Traumatology”[tw] OR “trauma”[tw] OR “traumas”[tw] OR polytrauma*[tw])

Scopus (advanced query string):

(TITLE-ABS-KEY(“x-ray” OR “x-rays” OR computedtomograph*) OR TITLE( “CT”)) AND (TITLE-ABS-KEY(“arm” OR “arms”)) AND (TITLE-ABS-KEY(“Whole Body” OR “total body” OR “Traumatology” OR “trauma” OR “traumas” OR polytrauma*)) AND PUBYEAR > 1998

## Appendix 2

**Table 6:** Quality score evaluated by the assessment criteria in Table 1. A maximum score of 7 points could be derived when an article meets all statements

Study	Selection		Outcome measurement		Comparability			Total points
	Subject inclusion criteria.	Selection of the non-intervention cohort.	Assessment of outcome.	Assessment of subjects.	Comparability of cohorts on the basis of the design.	Comparability on the basis of the cohort.	Comparability in CT settings.	
Beenen et al. [13]	✶	✶	✶	✶	✶		✶	6
Brink et al. [14]	✶	✶	✶	✶	✶	✶	✶	7
Heyer et al. [11]	✶	✶			✶			3
Hickethier et al. [15]		✶	✶	✶	✶		✶	5
Hoppe et al. [8]	✶	✶		✶	✶	✶	✶	6
Kahn et al. [9]		✶		✶	✶		✶	4
Karlo et al. [12]		✶	✶		✶		✶	4
Reske et al. [16]	✶	✶	✶	✶	✶		✶	6
Sedlic et al. [17]	✶	✶	✶	✶	✶			5

**Table 7:** Study characteristics (in alphabetical order)

Study	Year^a^	Study design	Country	No. of patients	Quality assessment^b^	Arm positions^c^
Beenen et al. [13]	2014	Single center prospective study	The Netherlands	30	6	A, B, C
Brink et al. [14]	2008	Single center retrospective study	The Netherlands	177	7	A, B, D
Hickethier et al. [15]	2018	Single center retrospective study	Germany	200	5	A, C
Hoppe et al. [8]	2006	Single center prospective study	Switzerland	83	6	A, D
Kahn et al. [9]	2013	Single center retrospective study	Germany	406	4	A, B, D
Karlo et al. [12]	2011	Single center retrospective study	Switzerland	150	4	A, C, D
Reske et al. [16]	2018	Single center retrospective study	Germany	308	6	A, D
Sedlic et al. [17]	2013	Single center retrospective study	Canada	67	5	A

^a^Year of publication

^b^Quality score evaluated by the assessment criteria in Table 1

^c^Arm positions, A: arm repositioning (arms elevated for abdomen CT and arms down for head and neck CT) and both arms elevated (alongside the head), B: one arm elevated and one arm either down, or in front of the upper abdomen, or placed in front of the pelvic area, C: arms on a pillow ventrally to the chest or upper abdomen and both arms crossed in front of the upper abdomen (without pillow), D: arms down (alongside the body) and both arms crossed in front of the pelvic area
